# Polyoxometalate on functionalized graphene sheets as a hybrid catalyst for efficient synthesis of benzimidazoles

**DOI:** 10.1038/s41598-025-91607-7

**Published:** 2025-03-06

**Authors:** Soghra Hossinimotlagh, Ali Zarnegaryan, Zahra Dehbanipour

**Affiliations:** Department of Chemistry, Yasouj University, 75918-74831 Yasouj, Iran

**Keywords:** Organic-inorganic, Hybrid catalyst, Benzimidazole, Graphene oxide, Catalysis, Chemistry

## Abstract

In this work, graphene oxide (GO) nanoparticles were synthesized and subsequently modified using 3-aminopropyltrimethoxysilane (APTMS). An Anderson-type polyoxometalate [(C_4_H_9_)_4_N]_2_[CrMo_6_O_18_(OH)_6_] was then immobilized on the surface of the modified graphene oxide nanoparticles. The obtained catalyst was characterized using Fourier-transform infrared spectroscopy (FT-IR), energy-dispersive X-ray spectroscopy (EDS), inductively coupled plasma (ICP), thermogravimetric analysis (TGA), scanning electron microscopy (SEM), Raman spectroscopy, and X-ray diffraction (XRD). The catalytic performance of this recyclable hybrid catalyst was evaluated for the synthesis of benzimidazole derivatives at 75 °C under solvent-based conditions. The hybrid catalyst demonstrated easy separation and could be successfully reused at least six times with only a slight reduction in the yield of the desired product. Leaching and recovery tests, along with FT-IR analysis, confirmed the high stability of the catalytically active species and the heterogeneous nature of the catalyst.

**Supplementary Information:**

The online version contains supplementary material available at 10.1038/s41598-025-91607-7.

## Introduction

Benzimidazoles and their derivatives are crucial building blocks due to their significant role in the pharmaceutical industry and their presence in bioactive natural products^1^. These compounds represent an important heterocyclic unit in various drugs, including antiviral, antiulcer, anticancer, antihistaminic, anti-HIV, antifungal, antihypertensive, antibacterial, anthelmintic, and antipsychotic agents^2–6^. The use of waste production, high energy consumption, toxic solvents and the application of traditional processes that do not follow the principles of green chemistry are problems for the pharmaceutical industry. The organic synthesis of chemical structures representing the starting point for obtaining active pharmacological compounds, such as benzimidazole derivatives, has become a focal point in chemistry. However, these techniques suffer from harsh reaction conditions (strong acids, high temperatures), prolonged reaction times, use of toxic reagents/catalysts, use of hazardous and toxic solvents, low yields, and formation of by-products^7^. The drawbacks above, make, it timely and highly desirable to develop more convenient and environmentally friendly protocols for synthesizing such biologically important compounds.

Polyoxometalates (POMs) exhibit reversible multi-electron redox properties and are well-established as high-efficiency catalysts due to their resistance to oxidative decomposition and excellent catalytic performance. These properties have made POMs widely applicable in various fields, including electrochemical functional materials^8,9^. Anderson-type POMs are characterized by a unique planar structure consisting of a central metal–oxygen octahedron surrounded by six edge-sharing MO_6_ (M = Mo or W) octahedra. Each addendum metal atom (Mo or W) in the skeleton is accompanied by two terminal oxygen atoms^10,11,12^. Despite their high catalytic activity and performance, the solubility of these compounds in polar or organic solvents presents challenges for catalyst recovery. To address this issue, polyoxoanions are often loaded onto various porous carriers to develop stable heterogeneous catalysts that mitigate the high solubility of POMs^13,14^. This strategy not only prevents catalyst leaching during the catalytic process but also reduces the risk of agglomeration.

Graphene is renowned for its unique and exceptional properties, including a large specific surface area, outstanding mechanical strength, excellent electrical conductivity, flexibility, thermal stability, and remarkable physical characteristics. These attributes form the foundation for its extensive research and applications in materials science and chemistry^15–18^. By functionalizing graphene with diverse chemical groups, various derivatives such as graphene oxide (GO) and fluorinated graphene can be produced, each offering unique properties. Graphene oxide, in particular, has attracted significant attention as a potential material due to its unparalleled features, including high adsorption capacity, large surface area, and chemical stability^19–21^. Graphene oxide, with its oxygenated groups and hydrophilic nature, is particularly suitable for functionalization. These unique structural characteristics make GO an excellent catalyst support, offering several advantages: First, the catalytically active species can be covalently immobilized on GO. Second, GO is cost-effective and readily available. Its amphiphilic nature and superb dispersibility in both organic and aqueous solvents make its surface highly accessible to various precursors^22,23^. Organic-inorganic hybrid catalysts supported on GO, with excellent recyclability, are promising candidates for green catalytic applications, combining environmental sustainability with practical efficiency^24–32^. However, many of the aforementioned systems suffer from issues such as catalyst leaching, instability, and low selectivity. These challenges can be attributed to the weak interactions between the catalytically active sites and the support material. Therefore, the preparation and design of efficient graphene oxide (GO)-supported catalytic systems that combine the advantages of both heterogeneous catalysts (easy recoverability) and homogeneous catalysts (high selectivity and activity) are of paramount importance in the field of catalysis. In light of these considerations, a novel and efficient graphene oxide-supported Anderson-type polyoxometalate (GO@CrMo_6_O_18_) was prepared and characterized. The catalytic activity of GO@CrMo_6_O_18_ was further investigated for the synthesis of benzimidazoles under specific reaction conditions.

## Experimental

### Synthesis of graphene oxide (GO)

Graphene oxide was synthesized following a published method^33^. Initially, concentrated sulfuric acid (12 mL), potassium persulfate (2.5 g), and phosphorus pentoxide (2.5 g) was stirred at 80 °C for 24 h. Subsequently, 3 g of graphite powder was added, and the mixture was stirred for 4.5 h at 80 °C for an additional 4.5 h, resulting in a slimy green mixture. After cooling to room temperature, 500 mL of deionized water was added, and the mixture was stirred for 2 h at room temperature. The reaction container was then left undisturbed for 24 h, after which the sediment was collected and dried.

### Synthesis of aminosaline functionalized graphene oxide (GO -APTMS)

**Fig. 1:**
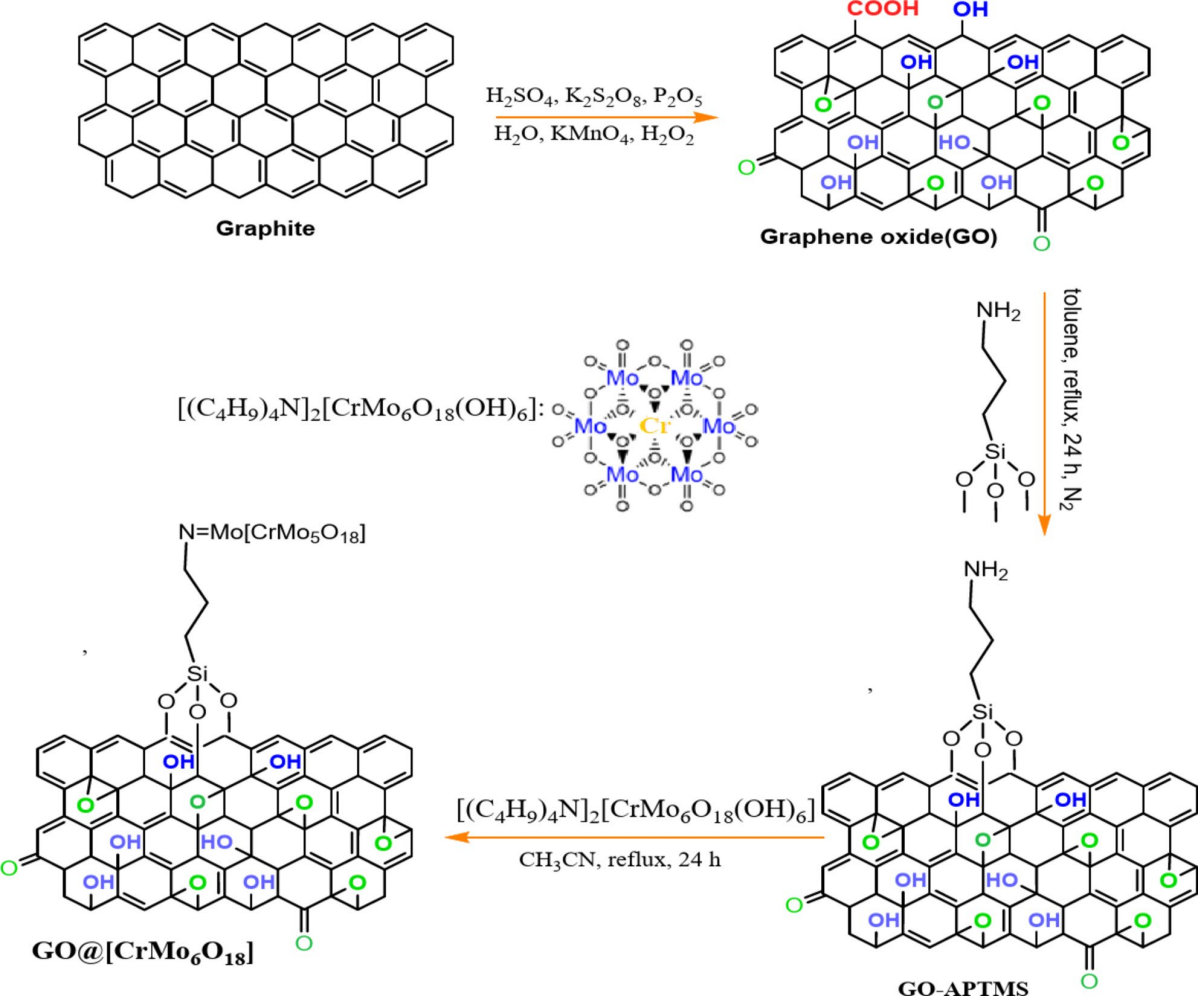
Ninhydrin test of GO-NH_2_.

In a typical preparation, 0.2 mmol GO was dispersed in 50 mL ethanol to form a suspension of GO nanoparticles. Subsequently, 1.2 mmol 3-aminopropyltrimethoxysilane (APTMS) was added to the suspension and the mixture was stirred under a nitrogen atmosphere at 60 °C for 7 h. The resulting black precipitate was filtered, washed with ethanol and toluene, and dried at room temperature. To confirm the presence of amino groups on GO-NH_2_, a ninhydrin test was performed as described in the literature^34^. After the test, the reaction solution turned black and exhibited a strong absorbance peak at 280 nm (Fig. 1). These results confirm that 3-aminopropyltrimethoxysilane was effectively immobilized on the surface of GO and that APTES@GO contains a large number of primer amine groups on its surface.

### Synthesis of [(C_4_H_9_)_4_N]_2_[CrMo_6_O_18_(OH)_6_

[(C_4_H_9_)_4_N]_2_[CrMo_6_O_18_(OH)_6_] was prepared according to a published procedure with slight modifications^35^. Initially, (C_4_H_9_)_4_N)_6_Mo_7_O_24_ .4H_2_O (5.0 g, 4 mmol) was dissolved in 80 mL water and stirred in an oil bath. Chromium (III) nitrate (2.3 g, 5.75 mmol) was separately dissolved in 20 mL of water and added dropwise to the solution, maintaining the pH within the range of 4 to 6.5 during the addition. After the addition was complete, the mixed solution was stirred at a constant temperature for 1 h. The resulting red wine-like solution was left undisturbed at room temperature for 12 h, leading to the precipitation of purple crystals. Upon recrystallization, filtration, and vacuum drying, pink crystals (4.9 g) were obtained.

### Synthesis of GO@CrMo_6_O_18_

A stirred solution of APTMS/GO (0.50 g) in CH_3_CN solvent (10 mL) was heated at 80 ℃. To this, a solution of [(C_4_H_9_)_4_N]_2_[CrMo_6_O_18_(OH)_6_] (0.50 g) in CH_3_CN (10 mL) was added. The reaction mixture was vigorously stirred under reflux conditions for 20 h. The resulting composite material was isolated by vacuum filtration, thoroughly washed twice with CH_3_CN and twice with ethanol (C_2_H_5_OH), and then dried at room temperature.

### General method for the preparation of benzimidazoles

A suspension of phenylenediamine (1 mmol), benzyl alcohol (1 mmol), and GO@CrMo_6_O_18_ catalyst (30 mg) in CH_3_CN (10 mL) was prepared and stirred in a round-bottomed flask. The progress of the reaction was monitored by thin-layer chromatography (TLC) using a solvent system of ethyl acetate and hexane (1:2). After completion of the reaction, the mixture was cooled to room temperature, and EtOAc/EtOH (4:1) was added. After completion of the reaction, the mixture was cooled to room temperature and EtOAc/EtOH (5:2) was added. The catalyst was separated by filtration and washed with EtOH (7 mL). After the solvent evaporation, the crude solid obtained was purified by silica gel column chromatography (Merck, 60 − 120 mesh, ethyl acetate: petroleum ether as an eluent) to afford the pure product.

## Results and discussion

### Characterization of GO@CrMo_6_O_18_

**Fig. 2:**
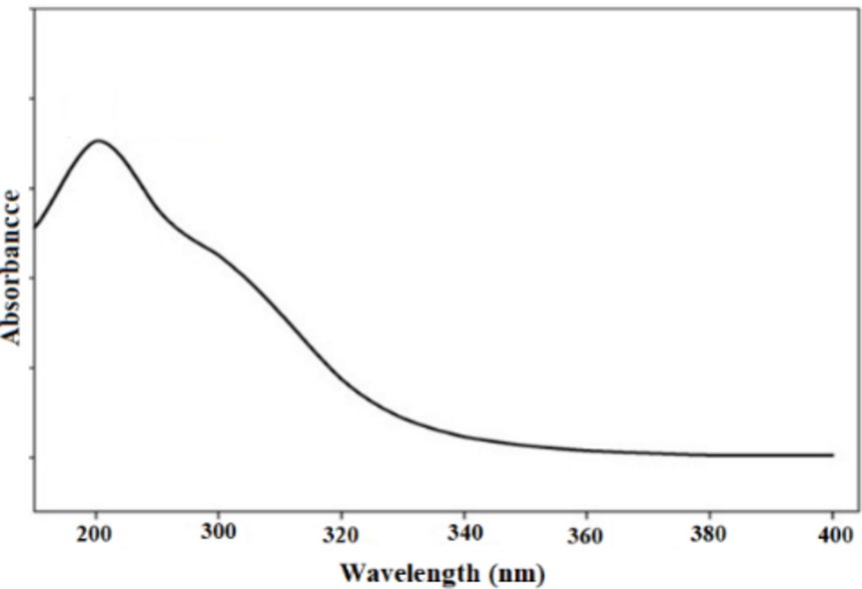
Synthetic strategy for GO@CrMo_6_O_18_.

The preparation process for GO@CrMo_6_O_18_ is illustrated in Fig. 2. Initially, GO and POM were synthesized according to previously reported procedures. The GO nanomaterial was then functionalized with APTMS to obtain GO/APTMS. Finally, the GO/APTMS support was treated with [(C_4_H_9_)_4_N]_2_[CrMo_6_O_18_(OH)_6_], resulting in the formation of the GO@CrMo_6_O_18_ nanocatalyst. It is noteworthy that the formation of GO@CrMo_6_O_18_ complex is achieved through a strong covalent interaction between the protonated amine groups of the GO/APTMS support and [(C_4_H_9_)_4_N]_2_[CrMo_6_O_18_(OH)_6_]. This interaction significantly enhances the stability and performance of the designed catalyst.

**Fig. 3:**
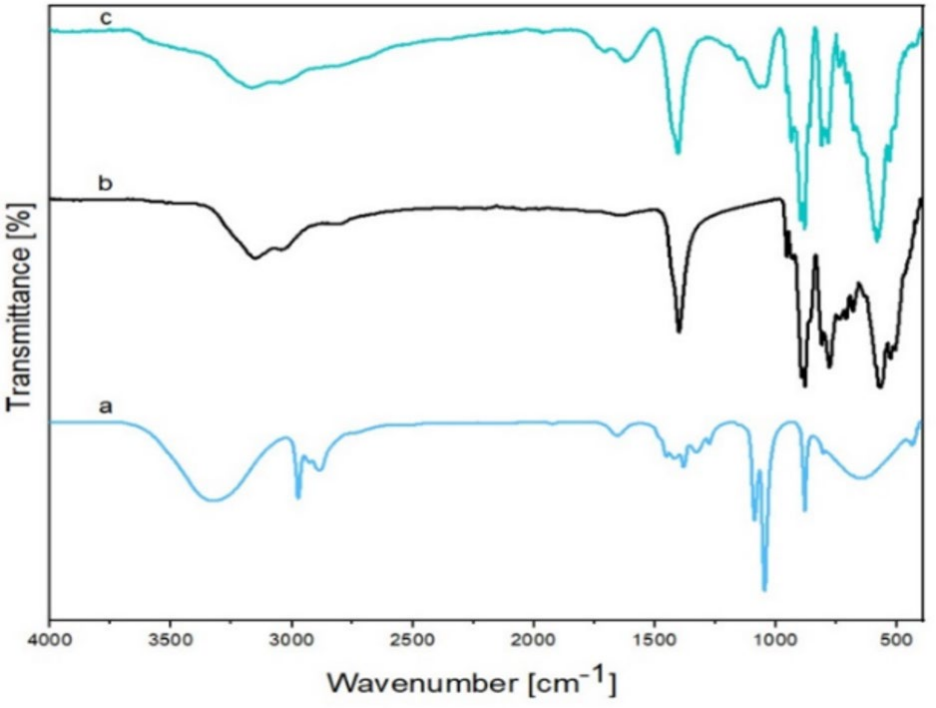
FT-IR spectra of (**a**) GO, (**b**) POM, and (**c**) GO@CrMo_6_O_18_ catalyst.

Fourier-transform infrared (FT-IR) spectroscopy was employed to identify the functional groups present in the prepared materials. (Fig. 3). The FT-IR spectrum of GO displays characteristic bands at 3381, 1721, 1624, 1226, and 1058 cm^–1^ (Fig. 3a) corresponding to the presence of O-H, C = O, C = C, C-O-C, and C-O, respectively^36^.

For the Anderson-type polyoxometalate, the FT-IR spectrum exhibit characteristic peaks (Fig. 3b) at around 932 cm^–1^ attributed to the Mo = O stretching vibrations, and 647 cm^–1^ corresponding to the Mo-O-Mo. The FT-IR spectrum of the GO@CrMo_6_O_18_ nanocatalyst exhibit signals at 2848 and 2941 cm⁻¹, which are attributed to the vibrations of the C–H bonds. Additionally, the peak at 1042 and 1131 cm⁻¹ correspond to the Si–O–C and the Si–O–Si bonds, respectively^37^. The presence of SiO₂ in the catalyst is indicated by the vibration bands at 3379 cm⁻¹, assigned to O–H vibrations, and 1063 cm⁻¹, corresponding to Si–O–Si vibrations. These observations confirm the incorporation of GO/APTMS particles and [CrMo_6_O_18_] cluster units in the synthesized nanocatalyst.

**Fig. 4:**
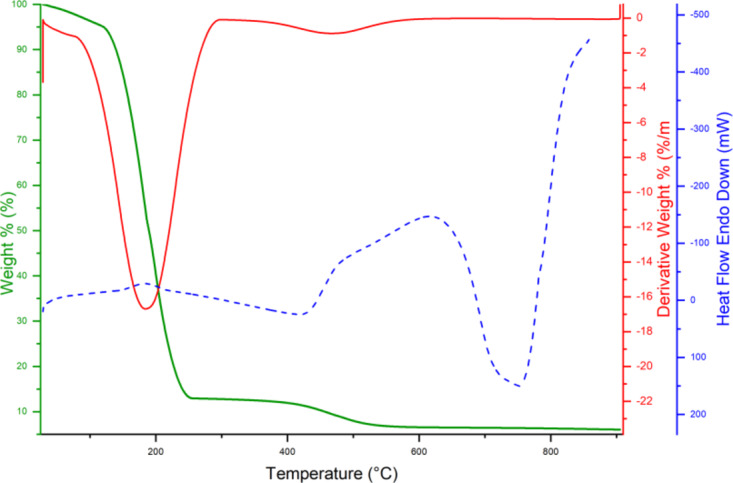
TG analysis of the GO@CrMo_6_O_18_ catalyst.

To evaluate the thermal stability, thermogravimetric analysis (TGA) was conducted at a heating rate of 10 °C/min in a nitrogen atmosphere (Fig. 4). The slight weight loss observed below 180 °C can be attributed to the evaporation of crystalline water, solvent molecules, and the decomposition of oxygen-containing functional groups on the GO surface^38^. In the first step, GO@CrMo_6_O_18_ showed a significant weight loss between 190 and 300 °C, which can be attributed to the removal of TBA counterions and organic groups from the polyanion. The subsequent gradual mass decrease observed in the plateau region (320–570 °C) corresponds to the decomposition of the polyanion into MoO_3_. In the second step, from 580 to 900 °C, the mass loss is likely due to the partial sublimation of MoO_3_, which is known to occur above 600 °C.

**Fig. 5:**
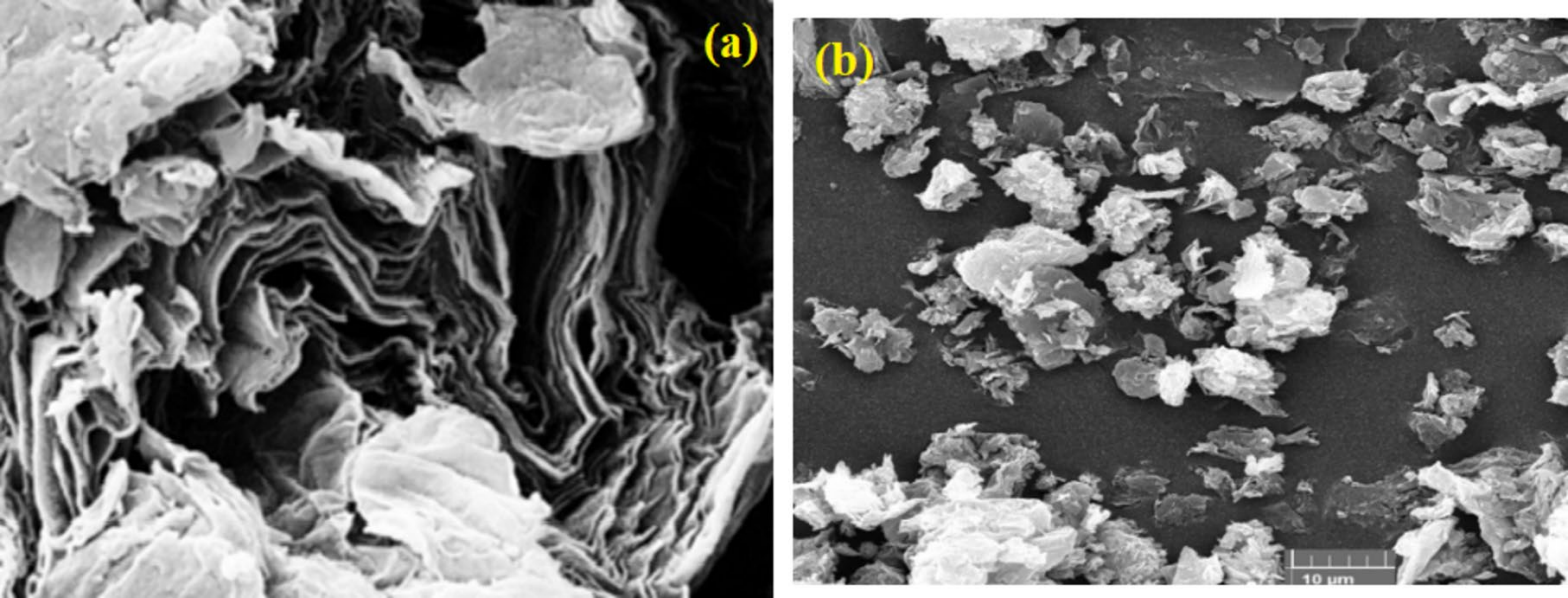
SEM images of (**a**) GO and (**b**) GO@CrMo_6_O_18_ catalyst.

The surface morphology of the GO and GO@CrMo_6_O_18_ materials was analyzed using scanning electron microscopy (SEM). The surface morphology of the GO and GO@CrMo_6_O_18_ materials was analyzed using scanning electron microscopy (SEM). The results indicate that GO exhibits a sheet-like shape with a wrinkled structure (Fig. 5a)^39^. After functionalization with the [(C_4_H_9_)_4_N]_2_[CrMo_6_O_18_(OH)_6_] complex, the lamellar structure of the GO@CrMo_6_O_18_ material remained intact, suggesting its potential as a highly efficient catalyst for organic transformations (Fig. 5b).

**Fig. 6:**
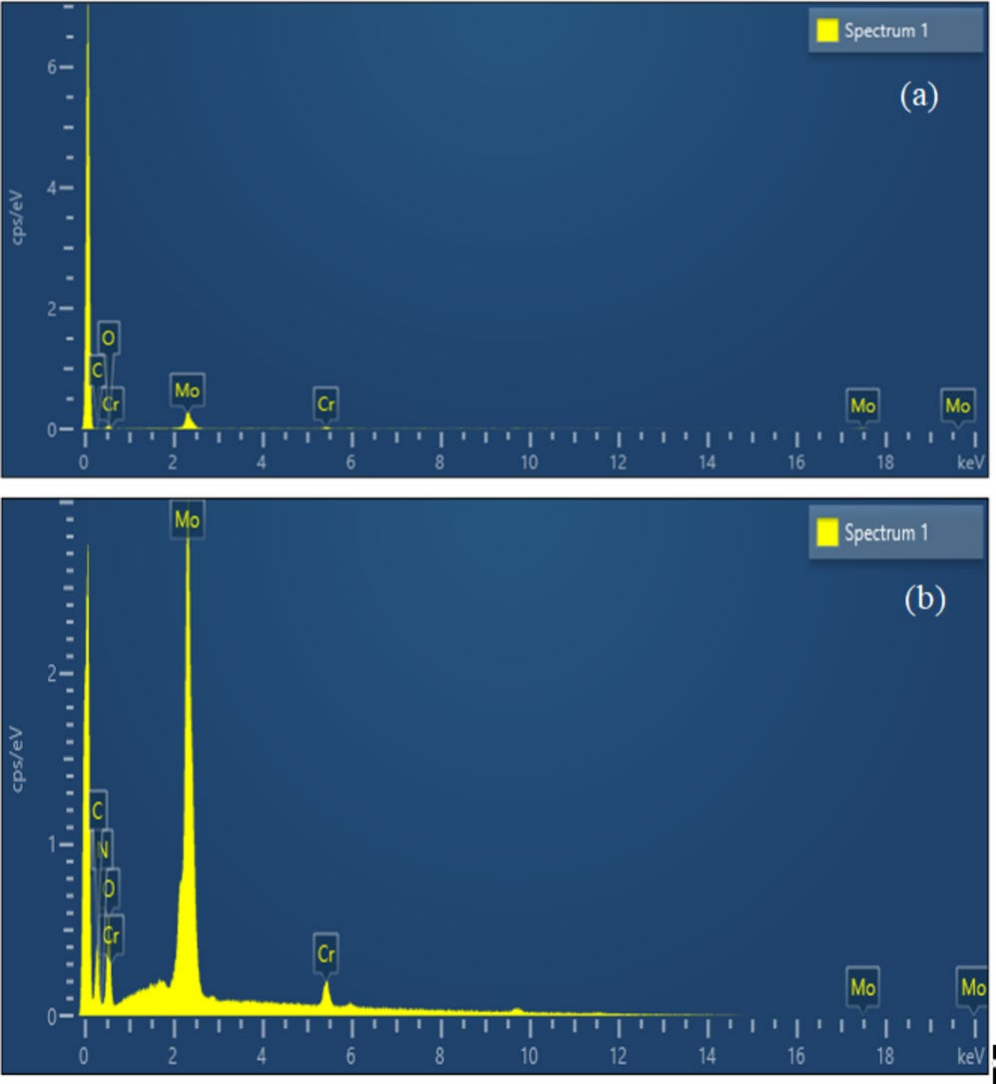
EDX results of (**a**) POM and (**b**) GO@CrMo_6_O_18_ catalyst.

**Fig. 7:**
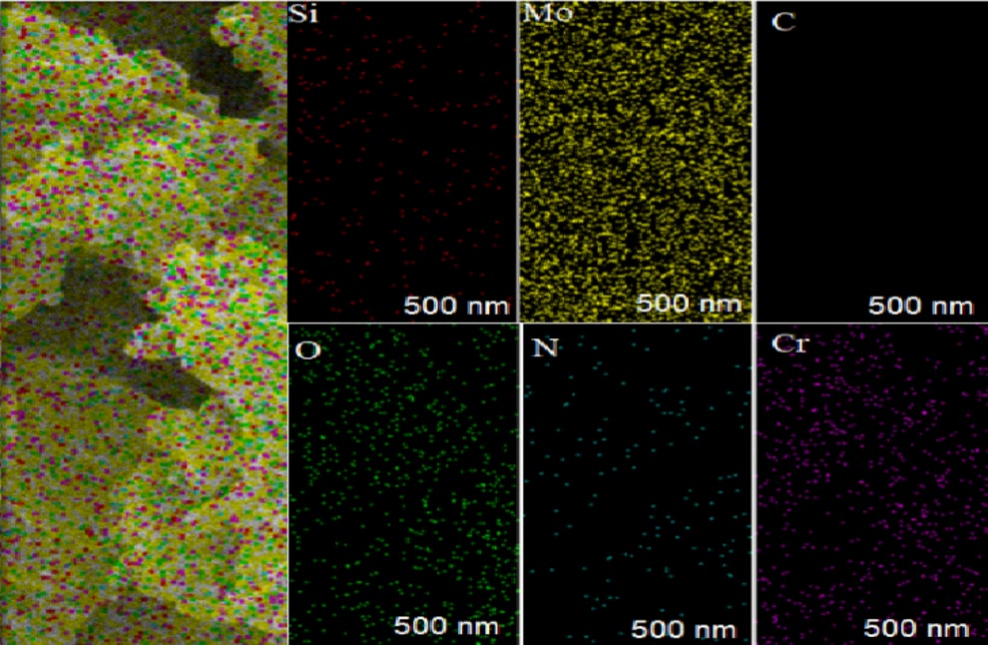
EDX mapping of the GO@CrMo_6_O_18_ catalyst.

Energy-dispersive X-ray (EDX) analysis was performed to investigate the distribution of the desired elements in the POM and GO@CrMo_6_O_18_ materials (Fig. 6). The presence of C, N, O, Cr and Mo elements in this analysis confirms the successful immobilization of the [CrMo_6_O_18_(OH)_6_] complex on the GO material, which aligns well with the FT-IR and TGA results. Additionally, EDX mapping confirmed that all the aforementioned elements were uniformly distributed throughout the GO@CrMo_6_O_18_ framework (Fig. 7).

**Fig. 8:**
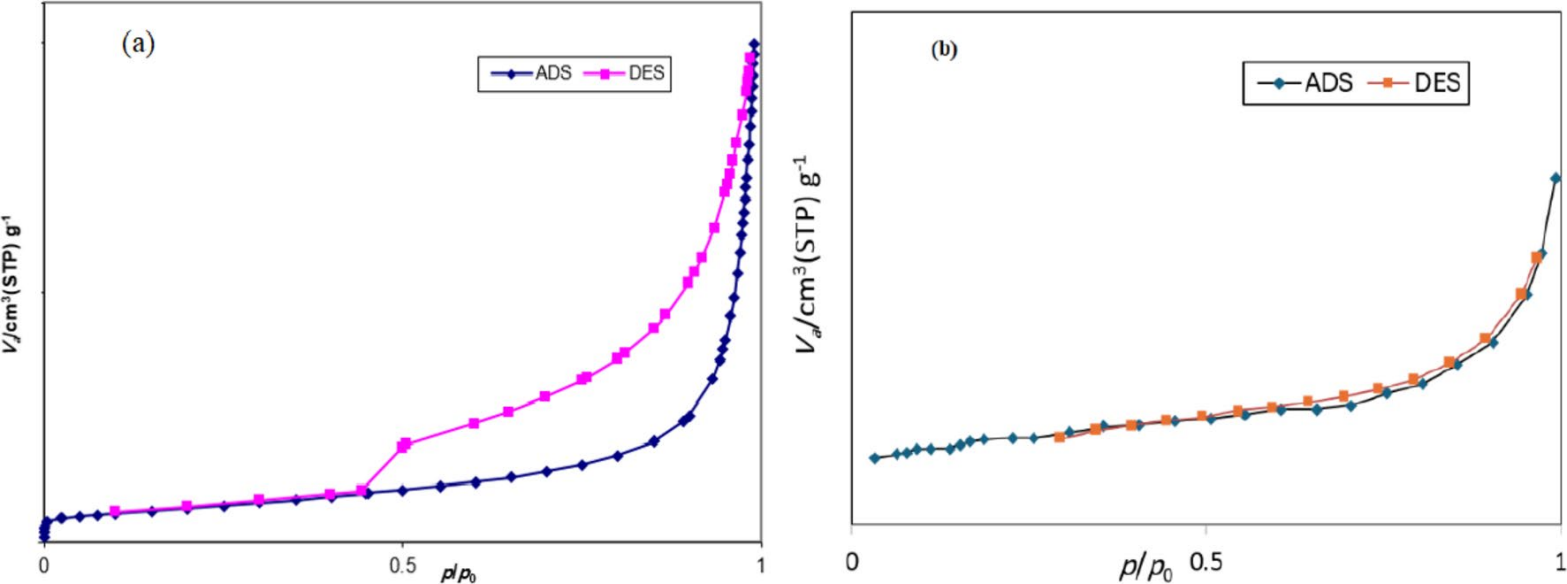
Nitrogen adsorption-desorption isotherms of (**a**) GO and (**b**) GO@CrMo_6_O_18_.

The nitrogen adsorption-desorption isotherms of GO and GO@CrMo_6_O_18_ catalyst are shown in Fig. 8. According to the IUPAC classification, the isotherm of GO to a type IV isotherm with an H_3_-type hysteresis loop, indicating a mesoporous material with layer structure. The specific surface area of GO before modification was 387.66 m^2^ g^–1^ and the average pore diameter was 25.36 nm. After surface modification and stabilization of the metal complex during the synthesis of the GO@CrMo_6_O_18_ catalyst, the surface area decreased to 12.94 m² g⁻¹. This reduction is expected due to the blockage of pores by the metal complex. Such changes confirm the successful immobilization of the anion ([n-Bu_4_N]_₂_[CrMo_6_O_18_(OH)_₆_]) on the surface of the GO nanosheets.

**Fig. 9:**
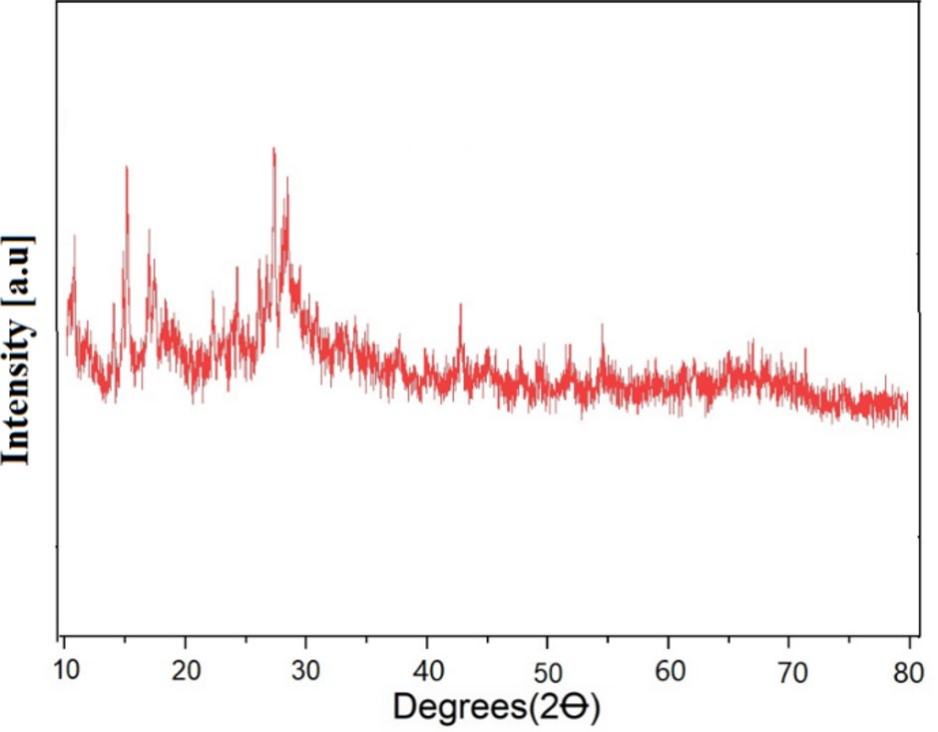
XRD patterns of GO@CrMo_6_O_18_catalyst.

The X-ray diffraction (XRD) pattern of GO@CrMo_6_O_18_ is exhibited in Fig. 9. In the GO@CrMo_6_O_18_ catalyst, in the XRD powder patterns of GO@CrMo_6_O_18_ a broad band centered at around 2θ = 29.58° is observed in the XRD powder patterns. However, in the case of GO@CrMo_6_O_18_, the peaks shifted to lower angles, owing to the support of the Cr complex and the increase the spacing between the nanosheets.

**Fig. 10:**
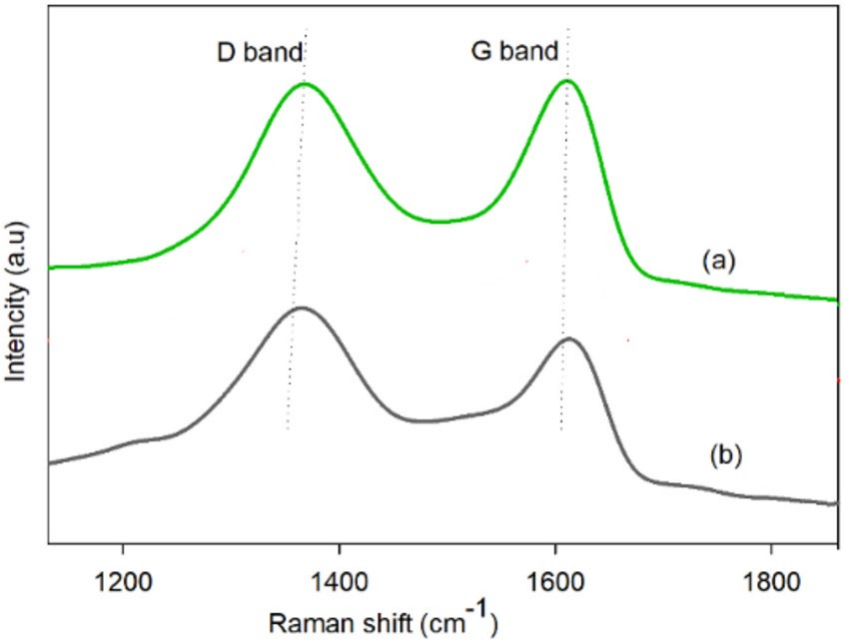
Raman spectra of the (**a**) GO and (**b**) GO@CrMo_6_O_18_ catalyst.

The Raman spectra of GO and GO@CrMo_6_O_18_ catalyst are shown in Fig. 10. The position of the D band is almost the same before and after the chemical modifications. However, the G band shifts from 1614 for GO to 1618 cm^− 1^ for GO@CrMo_6_O_18_ catalyst. This shift can be attributed to the attachment of the polyoxometalate to GO, causing an increased defect density in the graphene sheets.

### Effect of the catalysts on the synthesis of 2-phenyl benzimidazole

**Fig. 11:**
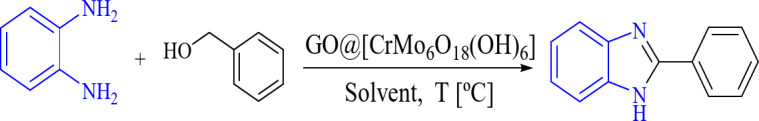
General method for the synthesis of benzimidazole derivatives using the GO@CrMo_6_O_18_ catalyst.

The catalytic activity of GO@CrMo_6_O_18_ was demonstrated in the synthesis of benzimidazole derivatives, as illustrated in Fig. 11.

The optimal reaction conditions were determined using phenylenediamine and benzyl alcohol as the model reactions (Table 1). Notably, the model reaction did not proceed in the absence of the GO@CrMo_6_O_18_ catalyst (Table 1, entry 1), highlighting the essential role of GO@CrMo_6_O_18_ in the synthesis of benzimidazole derivatives. As expected, the reaction proceeded in the presence of the catalyst and accelerated with an increased catalyst amount. Notably, when the catalyst amount was increased to 30 mg (corresponding to 1.58 mol% of chromium), the model reaction was completed within an acceptable timeframe (Table 1, entry 3).” Various solvents such as CH_3_OH, CH_2_Cl_2_, toluene, and CH_3_CN were evaluated. The study revealed that CH_3_CN provided the best results, achieving a high yield of 96% (Table 1, entry 3). Additionally, the effect of temperature on the model reaction was investigated, and the optimal results were obtained at 75°C with 1 mmol of phenylenediamine. As shown in Table 1, the highest yield, shortest reaction time, and best overall results were achieved using 30 mg of the catalyst in acetonitrile (CH_3_CN) at 75°C.

**Table 1:** Definition of the best reaction conditions for the synthesis benzimidazole in the presence of GO@CrMo_6_O_18_.

Entry	Amount of the catalyst	Solvent	Time (h)	Temperature [˚C]	Yield [%]^a^
1	–	CH_3_CN	4	75	N. R.^b^
2	20 mg, 1.26 mol%	CH_3_CN	3	75	87^c^
3	30 mg, 1.58 mol%	CH_3_CN	1.2	75	96
4	30 mg, 1. 58 mol%	CH_3_CN	2	75	83
5	30 mg, 1.58 mol%	Toluene	1.5	Reflux	74
6	30 mg, 1.58 mol%	CH_3_OH	1.5	Reflux	78
7	30 mg, 1.58 mol%	CH_2_Cl_2_	1.5	Reflux	Trace
8	30 mg, 1.58 mol%	CH_3_CN	1.2	60	41

^a^Isolated yield within 80 min. ^b^No reaction. ^c^Isolated by thin-layer chromatography.

To evaluate the catalytic activity of the supported CrMo_6_O_18_, the reaction of phenylenediamine with benzyl alcohol under optimized conditions was studied using GO nanoparticles, GO-APTMS, CrMo6O18, chromium (III) nitrate, and GO@CrMo_6_O_18_. The results were compared to the same reaction in the presence of GO@CrMo_6_O_18_ (Table 2). As shown in Table 2, functionalized GO nanoparticles, CrMo_6_O_18_, and chromium (III) nitrate did not yield satisfactory results for the synthesis of 2-phenylbenzimidazole. In contrast, GO@CrMo_6_O_18_ facilitated the reaction, achieving a yield of 96% in just 80 min.

**Table 2:** Synthesis of phenyl benzimidazole in the presence of GO nanoparticles CrMo_6_O_18_, chromium (III) nitrate salt, GO-APTMS, and GO@CrMo_6_O_18_.

Entry	Catalyst	Yield [%]^a^
1	GO nanoparticles	35
2	CrMo_6_O_18_	81
3	chromium (III) nitrate salt	68
4	GO-APTMS	29
5	GO@CrMo_6_O_18_	96

^a^Reaction conditions: phenylenediamine (1 mmol), benzyl alcohol (1 mmol), and catalyst (30 mg) in CH_3_CN solvent at 75 °C for 1.2 h.

The catalytic application of the GO@CrMo_6_O_18_ catalyst was further explored in the reaction benzyl alcohol with phenylenediamine other benzyl alcohol derivatives (Table 3). benzyl alcohol having electron-withdrawing or electron-donating groups at the para- meta- or ortho-position of the aromatic ring was investigated, and all corresponding benzimidazoles were synthesized in good yields.

**Table 3:** Synthesis of benzimidazole derivatives catalyzed by GO@CrMo_6_O_18_.


Entry	Substrate	Product	Time [min]	Yield [%]^a^	TOF (h^− 1^)^b^	Found M. P. (°C)
1			80	96	45.68	293–295
2			85	91	40.84	249–252
3			95	83	33.24	272–275
4			70	87	47.16	293–296
5			80	83	39.49	219–237
6			100	94	35.38	291–292
7			90	90	37.97	252–253
8			90	91	38.39	319–321
9			85	92	41.29	238–240

^a^Isolated yield. ^b^Turnover frequency [calculated by this equation: TON/time (h)], Turnover number [calculated by this equation: Yield (%)/Cat. (mol %)].

### Comparison of the catalyst

The advantages of the GO@CrMo_6_O_18_ catalyst over previous catalysts were investigated in the synthesis of 2-phenylbenzimidazole via the reaction of phenylenediamine and benzyl alcohol (Table 4). This was evaluated in terms of the reaction time and product yield. Notably, in this study, the synthesis of benzimidazoles was achieved with a short reaction time and an acceptable yield in the presence of the reusable GO@CrMo_6_O_18_ catalyst.

**Table 4:** Comparison results of the GO@CrMo_6_O_18_ with other catalysts for the synthesis of 2-phenylbenzimidazole.

Entry	Catalyst	Reaction conditions	Yield [%]	Ref.
1	BAIL gel	solvent-free, 130 °C, 5 h	89	^40^
2	PS–Fe–salen	EtOH, 70 °C, 20 h	90	^41^
3	K10- Zn^2+^	Water-methanol, rt, 24 h	93	^42^
4	Pd (II)Cl_2_-BTP@MNPs	DMF, TBAB, 80 °C, 1.5 h	94	^43^
5	H_2_SO_4_@HTC	EtOH, 80 °C, 1.5 h	88	^44^
6	Co-g-C_3_N_4_-imine/TiO_2_	CH_3_CN, 70 °C ,4 h	92	^45^
7	CoFe_2_-O_4_/Cu (OH)_2_	solvent-free, 20 min	90	^46^
8	Pd@PS	DMF, K_2_CO_3_, 130 °C, 20 h	92	^47^
9	GO@CrMo_6_O_18_	CH_3_CN, 75 °C, 1.2 h	96	This work

### Recycling ability and leaching study of the catalyst

**Fig. 12:**
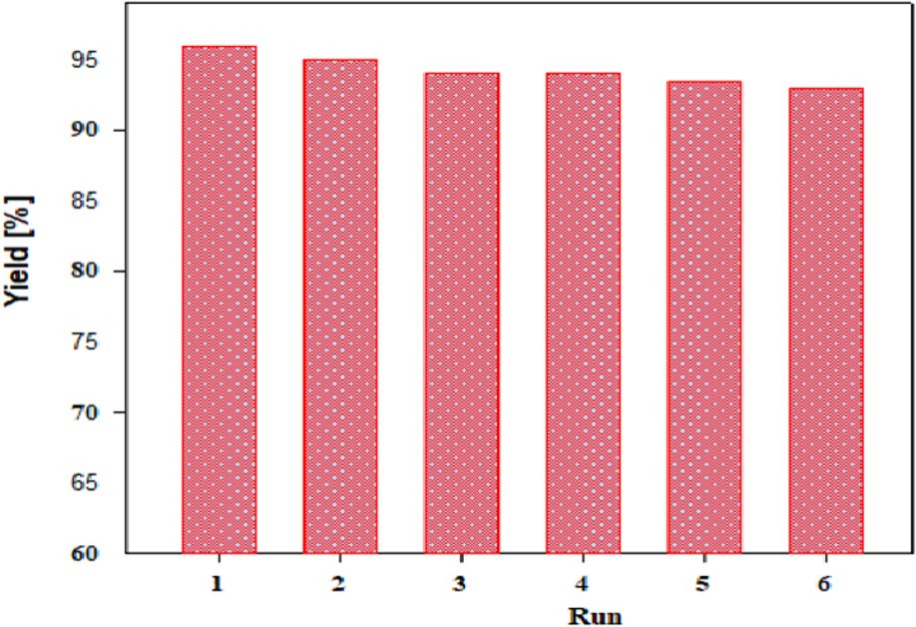
Reusability of the GO@CrMo_6_O_18_ catalyst.

TGA analysis revealed that the GO@CrMo_6_O_18_ catalyst remains at up to 300 °C. Consequently, its reusability was assessed in the synthesis of benzimidazoles via the reaction between phenylenediamine and benzyl alcohol. At the end of the reaction, the mixture was diluted and, the catalyst recovered by filtration and reused in the next run. As illustrated in Fig. 12, the GO@CrMo_6_O_18_ catalyst demonstrated effective recovery and reuse for up to six cycles. Chromium leaching from the catalyst during the synthesis of benzimidazoles was evaluated using a hot filtration test and ICP analysis. The reaction was repeated, and the catalyst was removed after 1 h. The reaction mixture was then allowed to proceed for an additional 2 h without the catalyst. No further reaction progress was observed, indicating that chromium was not leached under the reaction conditions. To confirm, the reaction was repeated, and the catalyst was removed by filtration at the end. The ICP analysis of the reaction solution showed no significant chromium leaching, reinforcing the catalyst’s stability during the process.

**Fig. 13:**
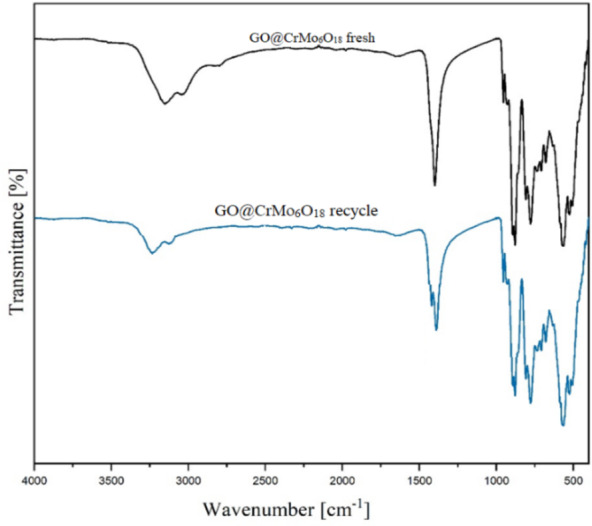
FT-IR spectra of GO@CrMo_6_O_18_ before and after the reaction.

The structural stability of the catalyst was evaluated after six cycles of use by analyzing the recovered catalyst using FT-IR spectroscopy. As shown in Fig. 13, the FT-IR spectrum of the recovered catalyst is identical to that of the fresh catalyst, confirming the structural stability of GO@CrMo_6_O_18_ throughout the recovery process.

### Plausible mechanism

**Fig. 14:**
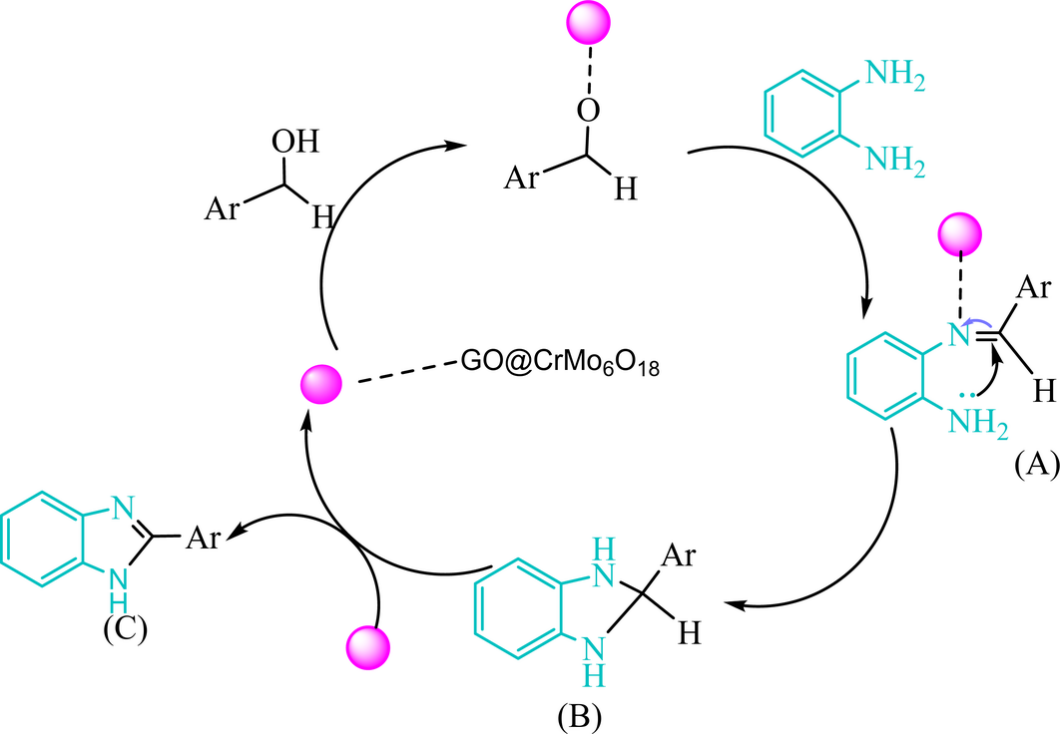
Proposed mechanism for the synthesis of benzimidazole in the presence of GO@CrMo_6_O_18_ catalyst.

A possible mechanism for benzimidazole compound synthesis from 1 to 2 diphenylamine with aromatic benzyl alcohol has been proposed and is illustrated in Fig. 14. Generally, the benzyl alcohol and an amine reaction proceed through the condensation reaction mechanism, forming a Schiff base and subsequently involving the oxidative cyclization reaction^48^. First, the activation of benzyl alcohol by the catalyst followed by condensation with the amino group of 1–2 diphenylamine results in imine (A), which upon the intramolecular nucleophilic attack of the remaining NH_2_ group in the presence of the catalyst affords intermediate (B). Finally, the oxidative aromatization of intermediate under ambient atmosphere in the presence of the catalyst afforded the desired product (C), releasing the catalyst for the next catalytic cycle.

## Conclusion

In summary, a novel graphene oxide-supported [(C_4_H_9_)_4_N]_2_[CrMo_6_O_18_(OH)_6_] complex (GO@CrMo_6_O_18_) was successfully synthesized, characterized, and applied as a highly efficient catalyst for the production of benzimidazoles. The FT-IR, TG, and EDX analyses confirmed the successful immobilization and high stability of the GO@CrMo_6_O_18_ complex within the material framework. Moreover, the SEM image demonstrated a well-defined lamellar structure for the catalyst. The GO@CrMo_6_O_18_ catalyst was effectively utilized for the synthesis of benzimidazoles under mild reaction conditions. It demonstrated excellent recyclability, being easily recovered and reused for at least six cycles without significant loss of catalytic activity. Additional advantages of this include its ability to operate at low temperatures, achieve high reaction rates, and require minimal catalyst loading. Further studies are currently underway in our laboratory to explore the potential application of this catalyst in other organic transformations.

## Electronic supplementary material

Below is the link to the electronic supplementary material.

Supplementary Material 1

## Data Availability

The datasets used and, or analyzed during the current study are available from the corresponding author upon reasonable request.
