# Supplementary material for: Polyoxometalate on functionalized graphene sheets as a hybrid catalyst for efficient synthesis of benzimidazoles
Source: Sci Rep. 2025 Mar 6;15:7880. doi: 10.1038/s41598-025-91607-7 (PMC11885448; doi:10.1038/s41598-025-91607-7)

**A novel organic****–inorganic** **hybrid catalyst of graphene oxide–polyoxometalate a highly efficient** **hybrid catalyst for the synthesis of** **benzimidazoles**

Soghra Hossinimotlagh, Ali Zarnegaryan*, Zahra Dehbanipour

*Department of Chemistry, Yasouj University, Yasouj, 75918-74831, Iran*

*E-mail: zarnegaryana@*[*yu.ac.ir*](mailto:salimibeni@yu.ac.ir)*, Tel.: (+98)-7431004128*

**^1^H and ^13^C NMR spectra of compounds**


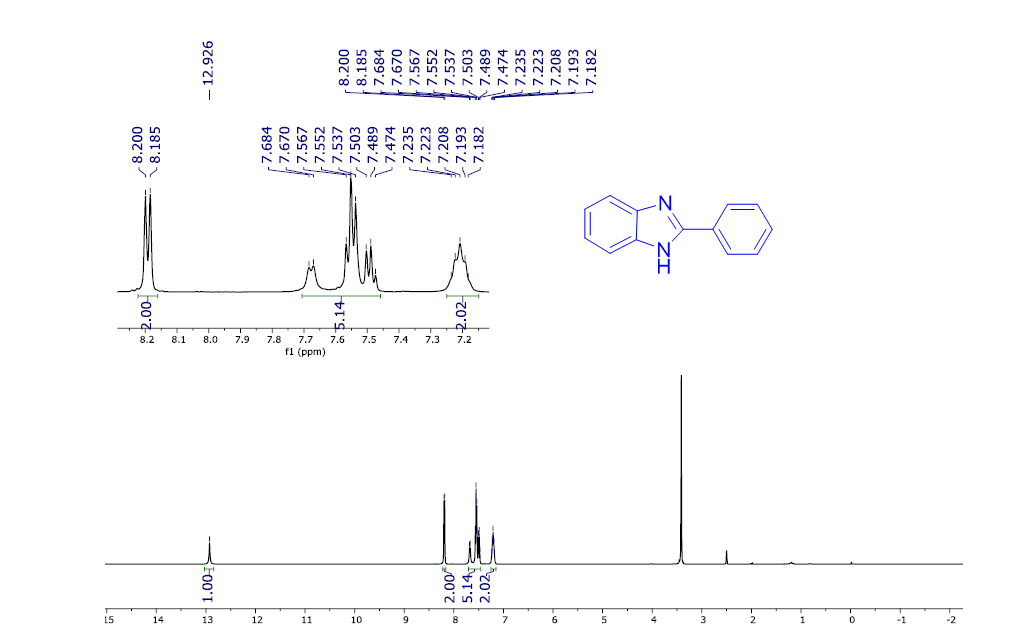


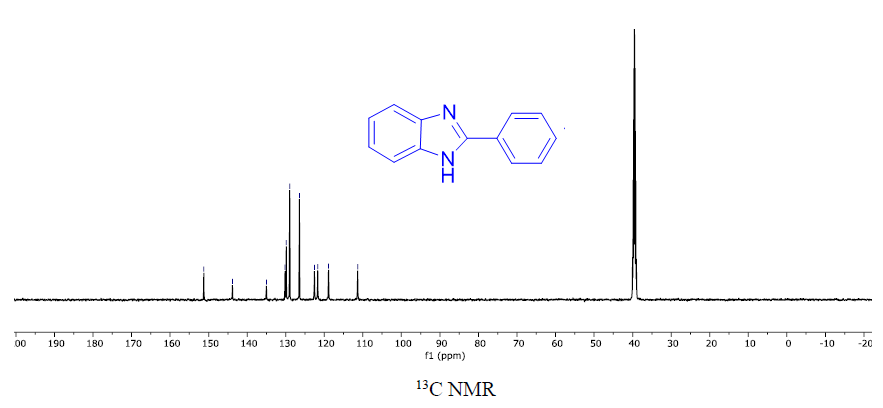


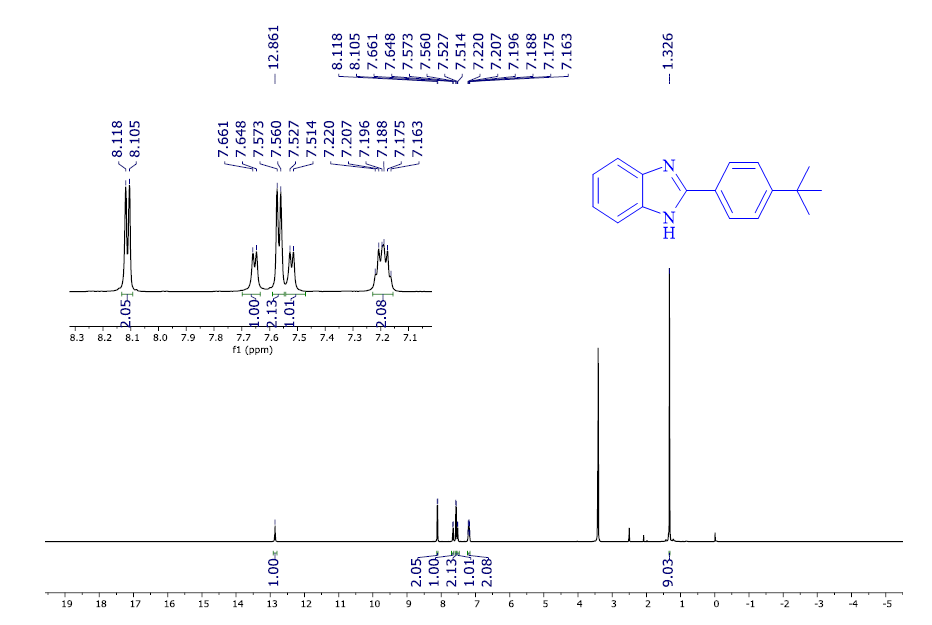


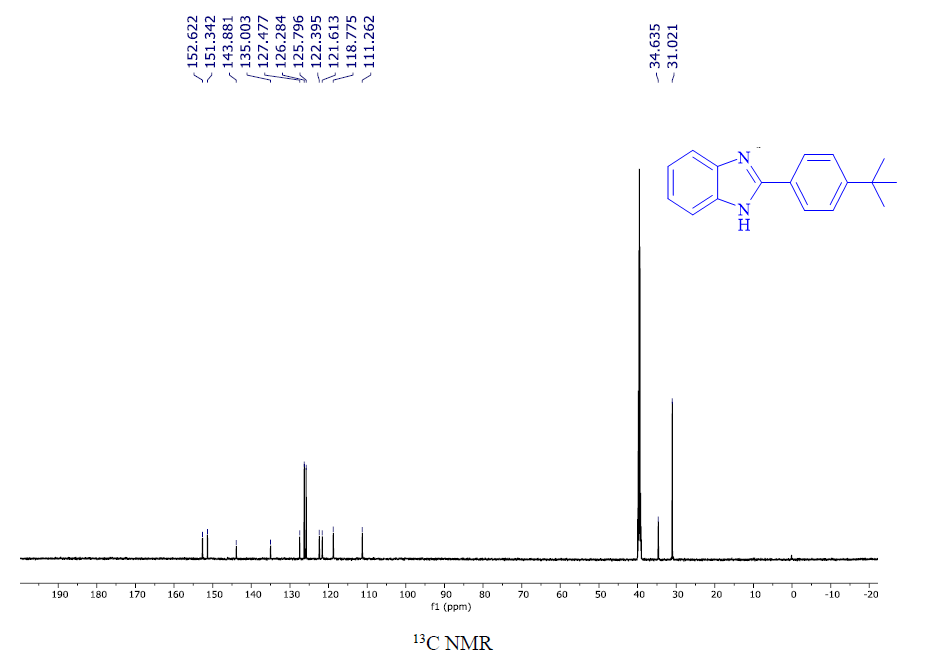


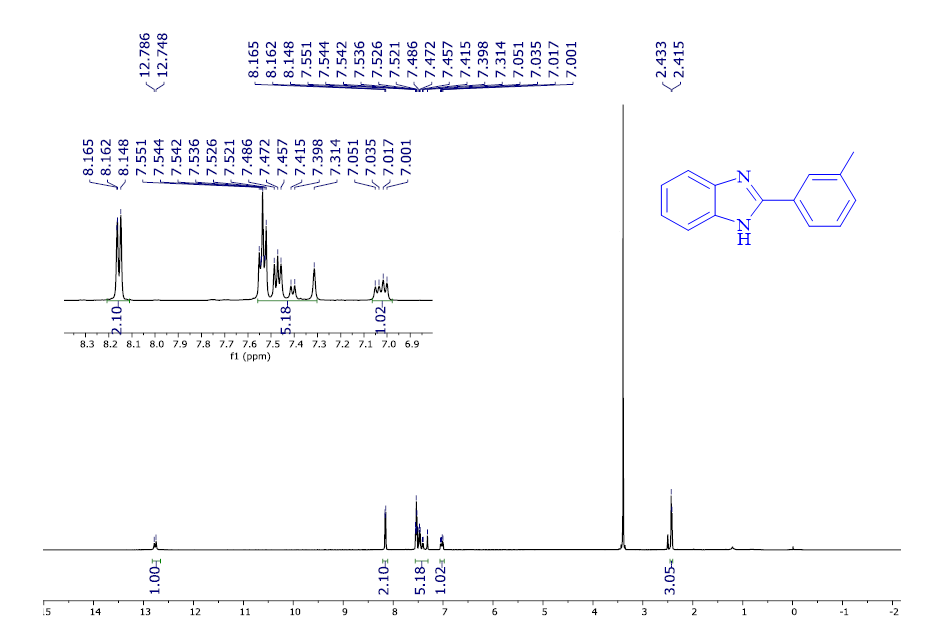


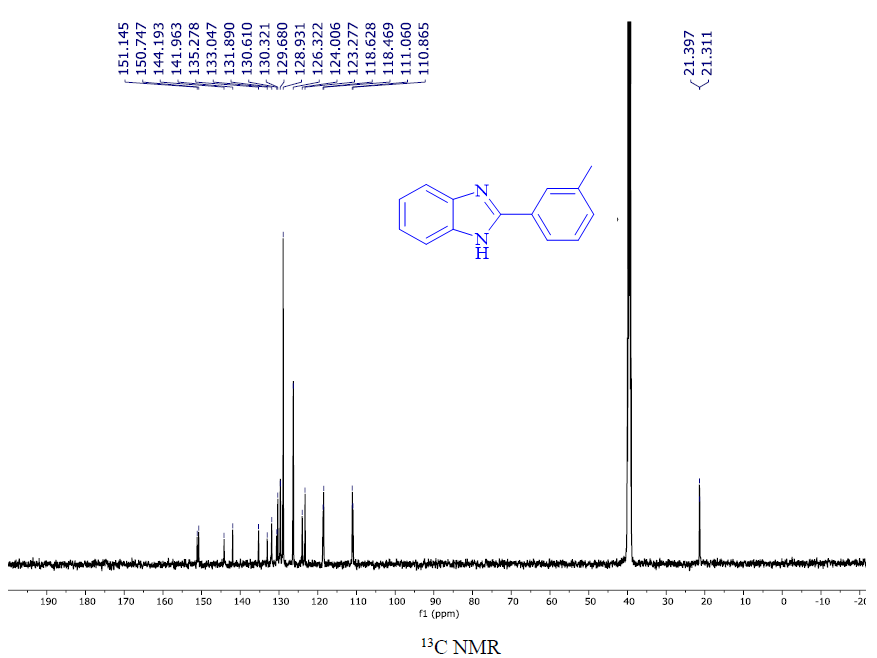


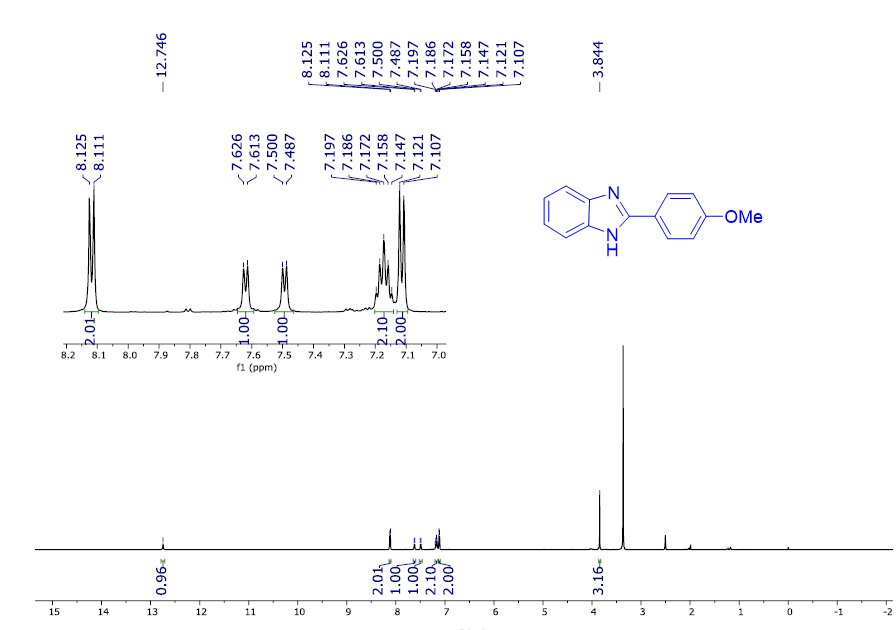


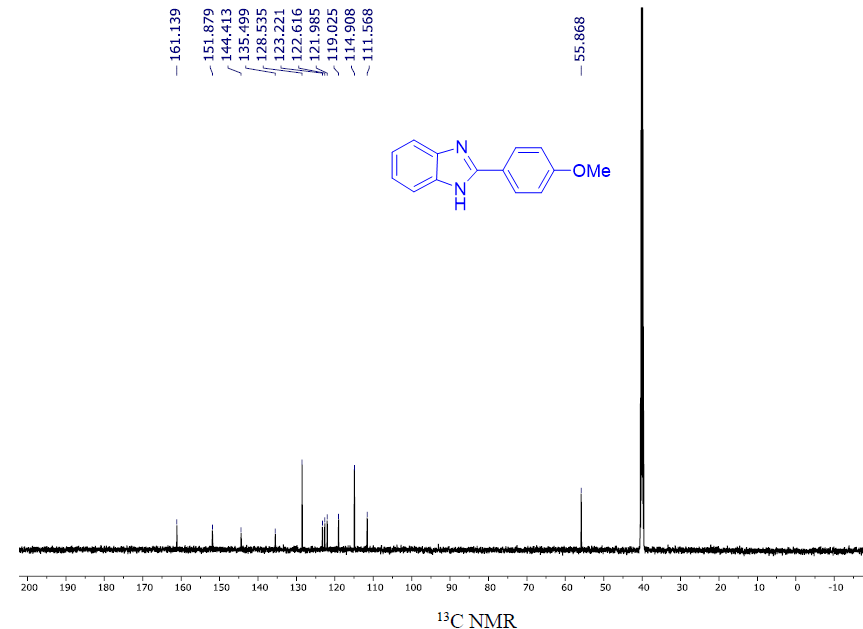


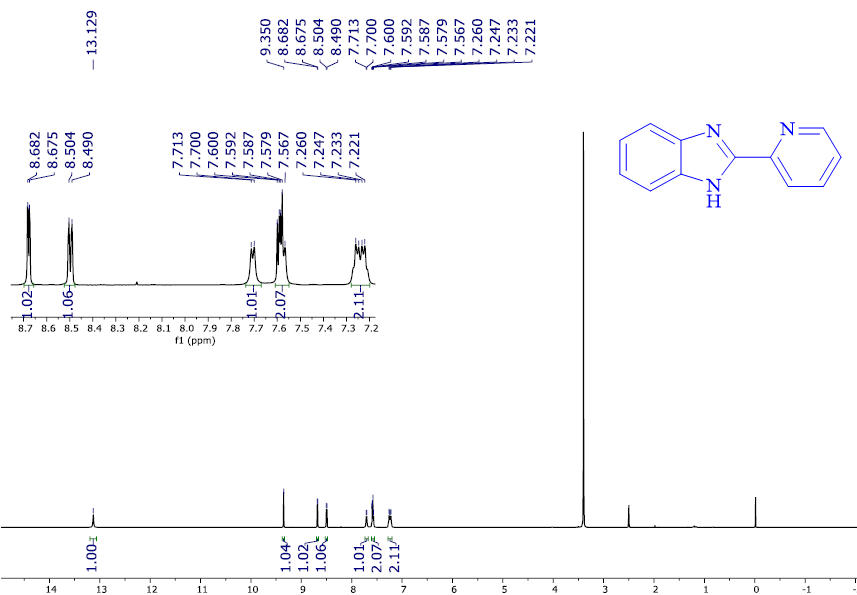


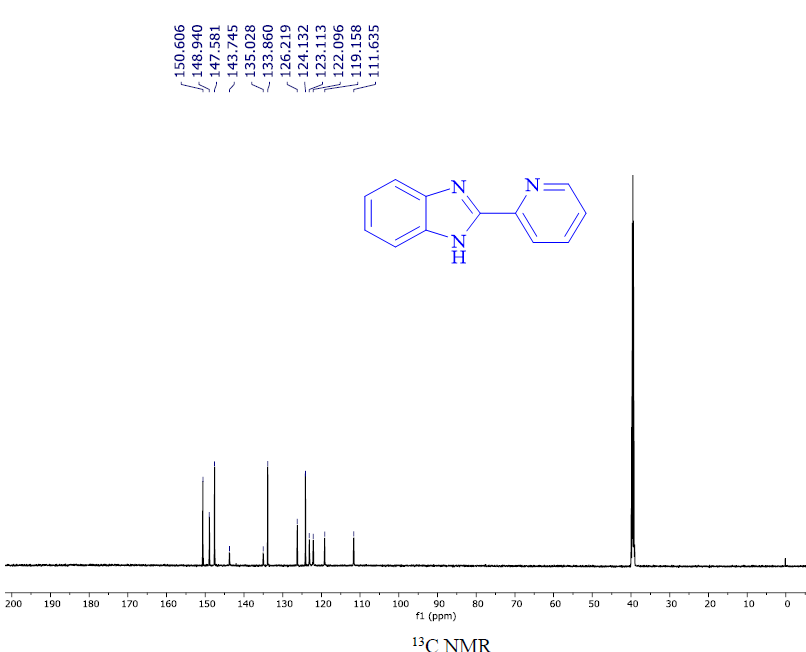


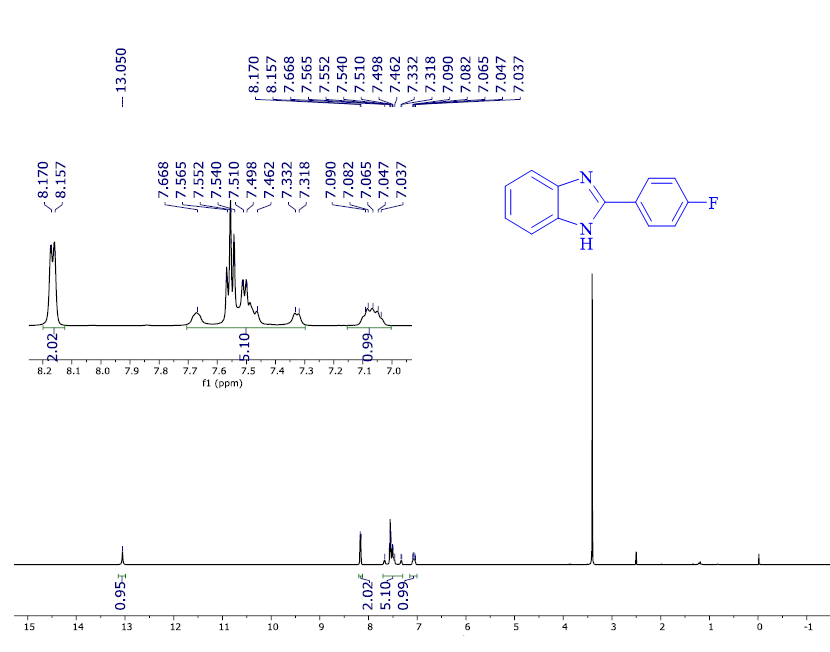


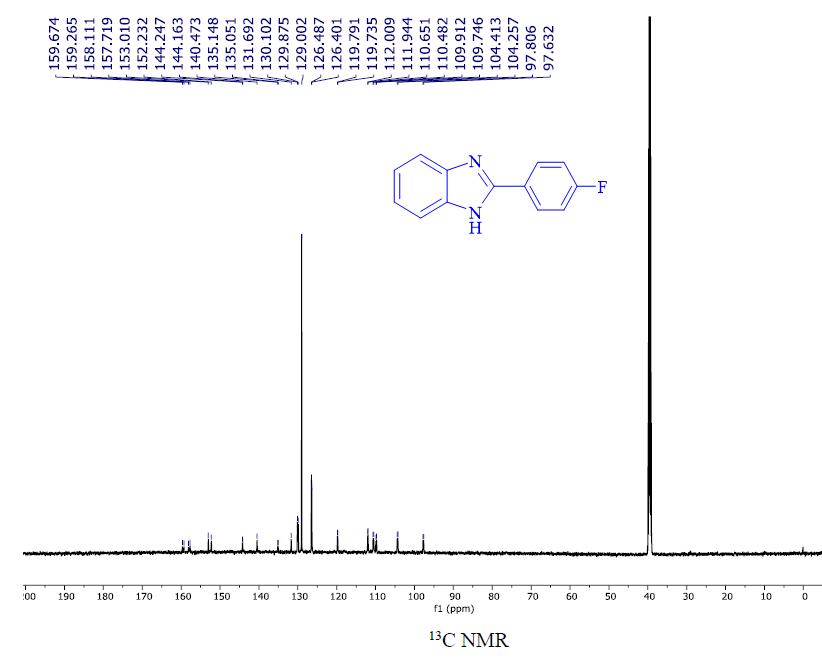


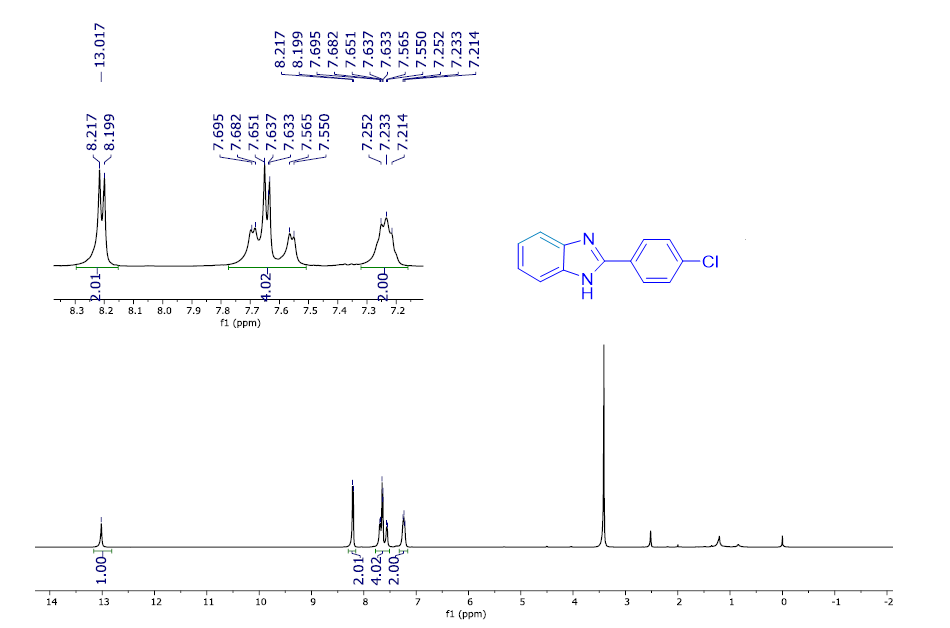

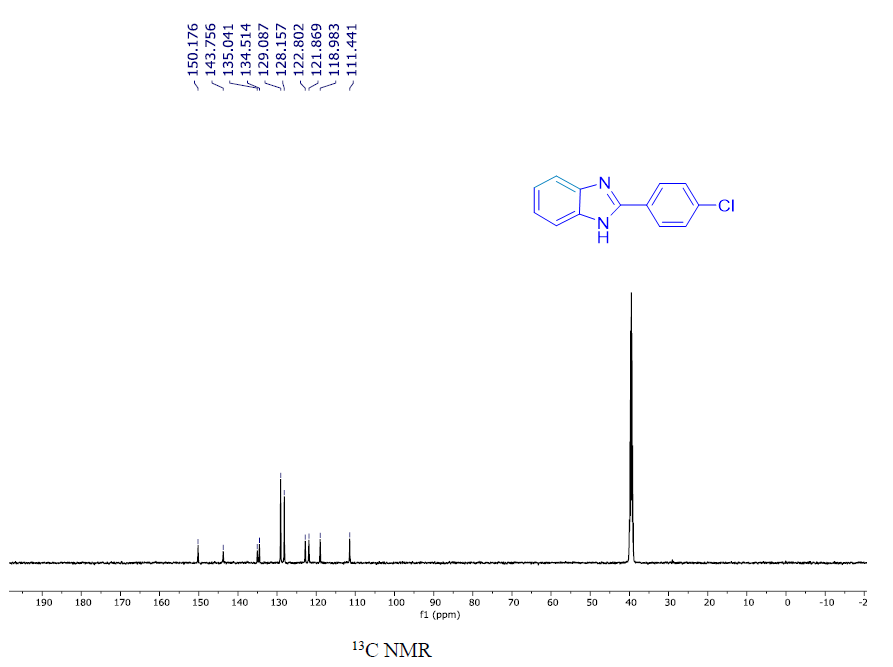


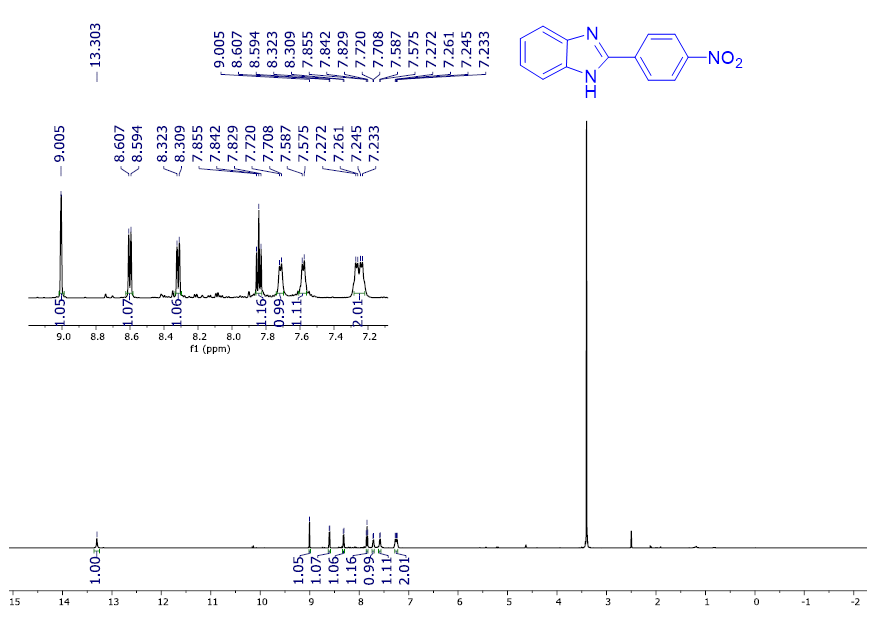


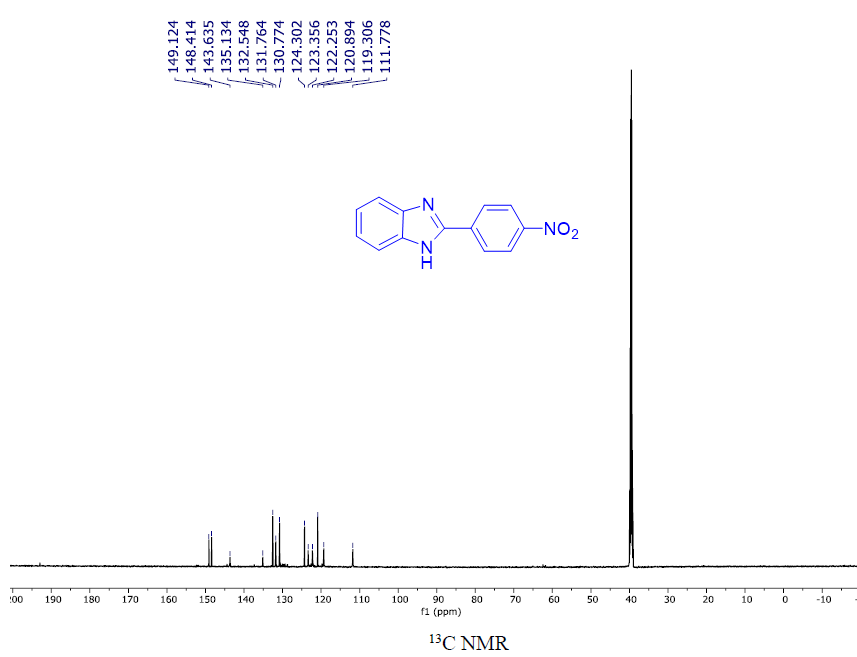


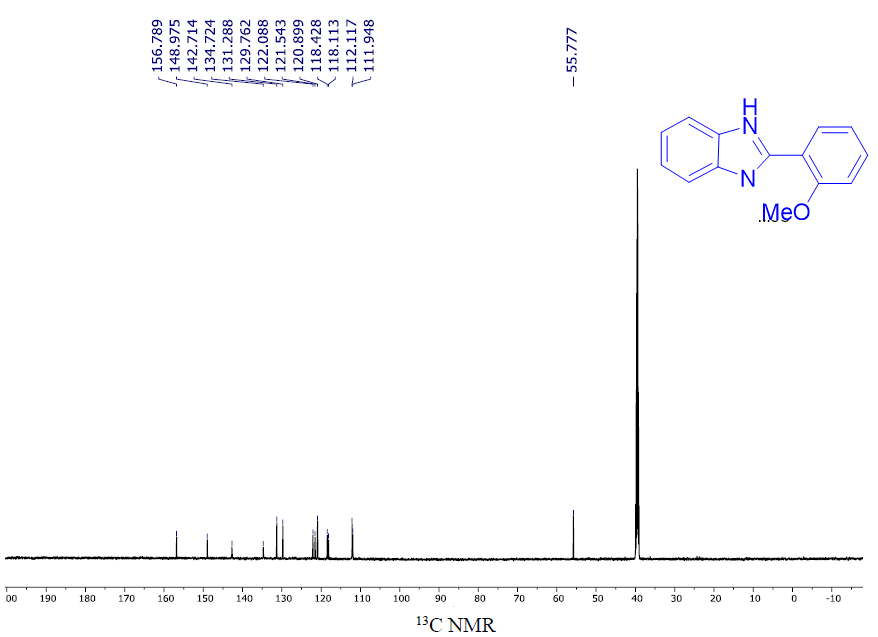

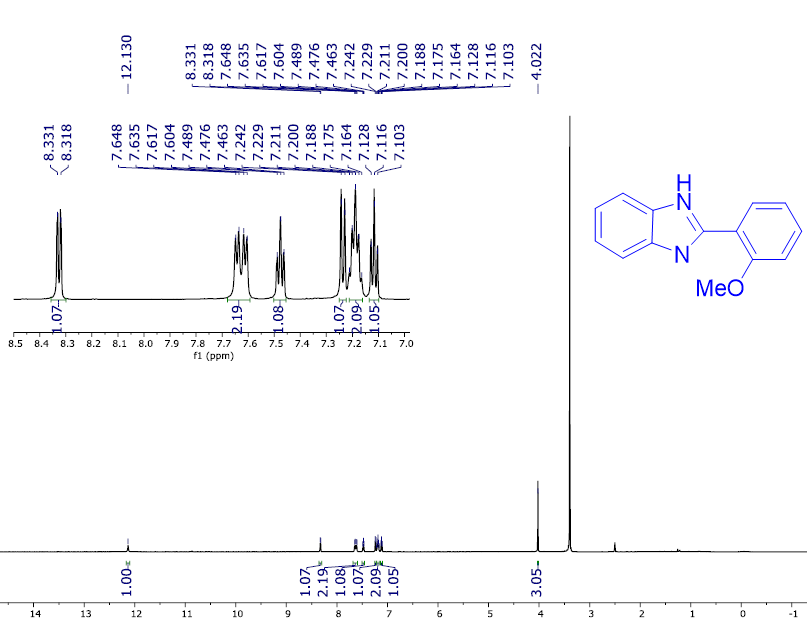

Supplement: Supplementary file 1 — Supplementary Material 1 [file 41598_2025_91607_MOESM1_ESM.docx]
